# Patient safety incidents in dentomaxillofacial imaging: reported adverse events from Hospital District Helsinki and Uusimaa and the City of Helsinki during 2012–2017

**DOI:** 10.1007/s11282-022-00616-z

**Published:** 2022-05-25

**Authors:** Marianne Suuronen, Taina Autti, Lasse Lehtonen

**Affiliations:** 1 Department of Dentomaxillofacial Radiology, HUS Medical Imaging Center, Helsinki University Hospital and University of Helsinki, Haartmaninkatu 4, 00290 Helsinki, Finland; 2 Department of Radiology, HUS Medical Imaging Center, Helsinki University Hospital and University of Helsinki, Helsinki, Finland; 3 HUS Diagnostic Services, Helsinki University Hospital and University of Helsinki, Helsinki, Finland

**Keywords:** Patient safety, Dentistry, Radiology, Risk management

## Abstract

**Objectives:**

Our study aimed to reveal the frequency of patient safety incidents (PSI) in dentomaxillofacial radiology (DMFR), including their mitigating and contributing factors, to help recognize and thus better prevent these adverse events (AE) in the future.

**Methods:**

Hospital District Helsinki and Uusimaa (HUS) and the City of Helsinki (HKI) use HaiPro, an anonymous web-based tool, for healthcare professionals to report PSI. Dentistry-related PSIs were evaluated individually to find any DMFR-related reports. Additionally, we searched the HaiPro-data using multiple dentistry- and DMFR-related keywords. We compartmentalized all DMFR-related PSI by their type and assessed their contributing factors, as well as their risk classification, severity, outcome, and possible corrective actions.

**Results:**

In HUS and HKI, 43 of the 195,589 HaiPro-reports filed during 2012–2017 were DMFR-related. The most prevalent event type of DMFR-related PSIs was laboratory-, medical imaging-, or other patient examination-related events (33%). The second most common event type was defined as being related to flow or control of information (26%). For both of these event types, the most common contributing factors were shortcomings of communication and flow of information. Risk classification showed only one AE to be of moderate risk, and all others were perceived as irrelevant or minor.

**Conclusions:**

PSI in DMFR are only rarely reported, and mostly, they are perceived of causing little or no harm. We detected a great difference in reporting activity between primary and secondary healthcare workers, but the underlaying causes remain unclear.

## Introduction

Patient safety in medical care has become a hot topic of research, particularly since the publication of To Err is Human in 2000 [1]. More recently, this patient safety focus has broadened to dentistry, including dentomaxillofacial radiology (DMFR). The role of imaging devices related to patient safety incidents (PSI) in dentistry, however, remains sparsely studied. Most device-related, safety-based studies in DMFR are about different dental materials and their compatibility with MRI, focusing on the different magnetic flux densities of 1.5 T, 3 T, and 7 T [2–4].

In contrast, many radiation protection studies in DMFR have been reported. Some of these note that different features of two-dimensional (2D) imaging devices have a positive effect on patient safety by providing diverse imaging protocols, better shielding options, and advanced technical features [5, 6]. All of these attributes, either by themselves or when used according to the basic radiation protection principle ‘ALARA’ (as low as reasonably achievable), reduce patients’ radiation dose. In addition to these new, more advanced conventional panoramic and cephalometric imaging devices, the introduction of three-dimensional (3D) cone beam computed tomography (CBCT) to dentistry in the late 1990s has had an indisputable impact on DMFR. CBCT provides great versatility to oral radiology, which explains the steady rise in the number of dental CBCT devices all over the world, including Finland [7–9].

Reporting patient safety incidents (PSI) related to medical devices and materials is similar to other European Union member states [10–12]. In Finland, these adverse events (AE) were previously reported to the National Supervisory Authority for Welfare and Heath (Valvira), but its operations concerning medical devices have been transferred to the Finnish Medicines Agency (Fimea) at the beginning of 2020 [13]. In Finland, voluntary reporting of other PSI is strongly advised and the most widely used tool for this is HaiPro. HaiPro is an anonymous web-based tool intended for healthcare professionals to report PSI. It is used by over 200 social service and healthcare organizations varying from small healthcare centers to entire hospital districts, and both Hospital District Helsinki and Uusimaa (HUS) as well as the City of Helsinki (HKI) use it [14].

**Fig. 1:**
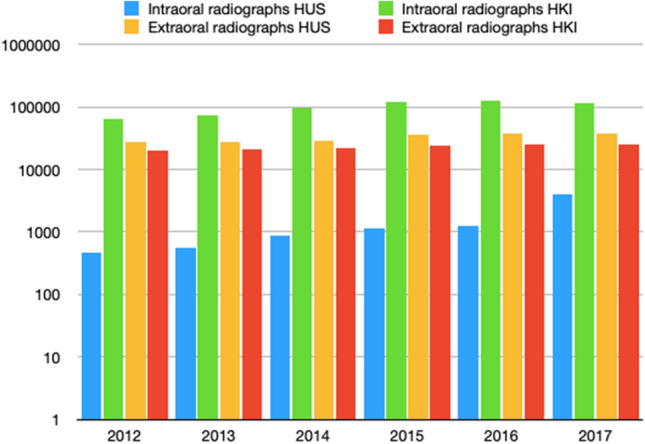
Amount of dental radiographs taken during 2012–2017: the rapid, almost threefold, increase in the number of intraoral radiographs from 2016 to 2017 (HUS) is due to the organizational change in which the dental students’ clinical practice was transferred from HKI to HUS

HUS covers the Finnish capital region and multiple other municipals of Southern Finland, with approximately 1.55–1.65 million inhabitants during 2012–2017, while HKI had approximately 600,000–650,000 inhabitants [15–18]. In HUS and HKI during 2012–2017, a total of 931,094 dental radiographs were taken, of which 201,113 (22%) were taken in HUS and 729,981 (78%) in HKI (Fig. 1). The large difference in these numbers is mainly because HUS provides secondary care services, while HKI provides primary care services.

Although dental imaging has a long history, data on patient safety incidents in dentomaxillofacial imaging are minimal both at the national and international levels. Thus, their contributing and mitigating factors remain unclear, which calls for research on the subject worldwide. The lack of identifying and reporting patient safety incidents or adverse events that are related to DMFR can also hinder healthcare organizations to achieve and maintain a high level of patient care quality.

The aim of this study was: (1) to identify and categorize PSIs in DMFR as well as their outcome in Southern Finland; (2) to evaluate how well these adverse events are detected and reported by healthcare workers; and (3) to determine whether the rapid increase in CBCT imaging has had any effect on the PSIs.

## Materials and methods

Finnish national law does not require ethics committee approval for registry studies with no patient intervention involved [19], but the required permission from the Research Administration of HUS and HKI was obtained for this study. We gained access to the whole HaiPro-data from HUS (HUS-data), but the HaiPro-data from HKI (HKI-data) were restricted to reports related to dentistry and reports related to diagnostics, which consisted of medical imaging and laboratory services. Notifications related to medical imaging were unavailable in the same matter in the HKI-data as in the HUS-data, because medical imaging was not listed as its own entity in the HKI-data. In HKI-data, imaging-related reports were combined with laboratory-related reports under the heading “Diagnostics.” Oral healthcare was implemented as its own entity in both data systems since the beginning of 2015.

HaiPro, the patient safety incident report, consists of multiple fields that specify the recognized PSI and its circumstances, including the department of the person filling the report, the department where the incident happened, and the occupational role of the reporter. Occupational roles include nurses, doctors, midwifes, and many more. The report also contains fields for the date and time of the incident, the nature of the incident, incident type, and description of the incident and its possible contributory factors. The nature of the incident can be defined as a near miss, as an actual incident or as another observation related to patient safety. The incident type is selected from a list of choices clarifying whether the incident is related to medication, flow, or control of information, to a diagnosis, a machine or its usage, or unknown—just to name few. The outcome and its severity to the patient, as well as to the department where the incident happened, and a risk classification of the event are also estimated by the reporter. Additional fields are for information about the measures taken, as well as for suggestions to prevent similar incidents in the future. The classification criteria of the HaiPro-system are similar—yet not identical—to the International Classification for Patient Safety (ICPS) published by the World Health Organization (WHO) [20].

**Fig. 2:**
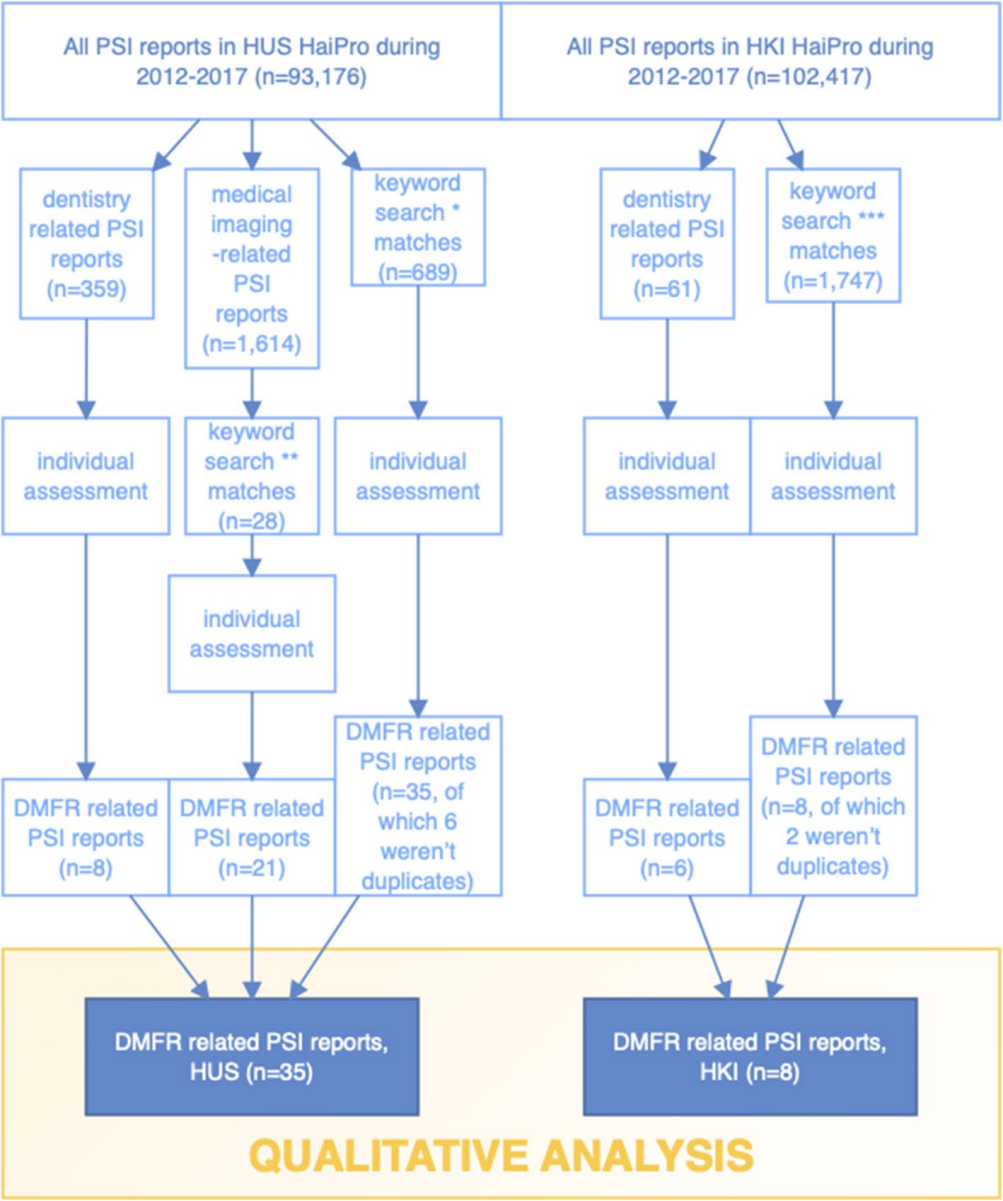
The used keywords and abbreviations are found separately listed and marked with corresponding asterisks in Table 1

**Fig. 3:**
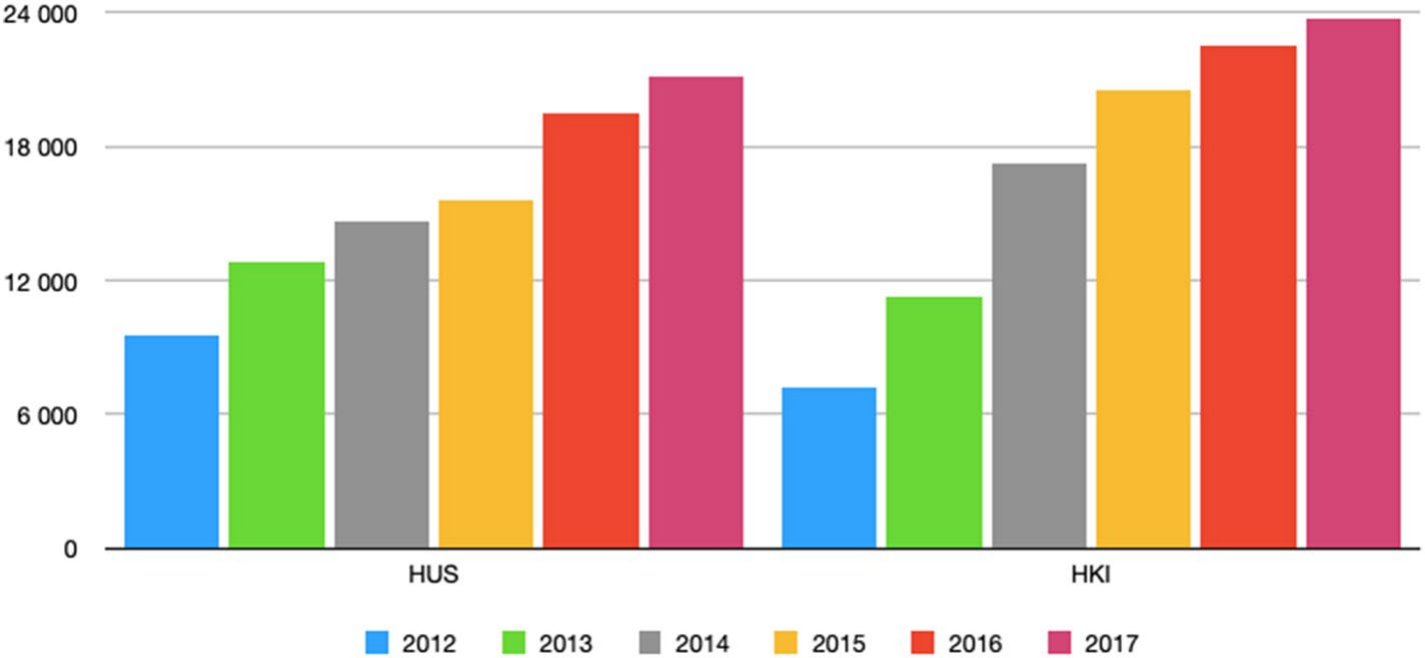
The amount of all HaiPro-reports made during 2012–2017 shows a steady growth during the evaluation period both in HUS and HKI

We searched both databases for the reports filed in 2012–2017 using the search tool integrated in the HaiPro-system. The text search tool can search for keywords or abbreviations through either the whole database or under preset headings. We used multiple keywords and abbreviations consisting of the most commonly used Finnish terms in dentistry and DMFR, which are listed in Table 1. The differences between the chosen keywords in both data systems were due to the use under preset headings and the aforementioned structural differences in the HUS-data compared to the HKI-data. Additionally, all dentistry-related PSI reports, as well as those found with the keyword search, were evaluated individually to avoid missing any DMFR-related reports. Due to our partly keyword-based and partly manual screening-based search, duplicates were found and excluded. Keyword search revealed cases that were not related to DMFR, e.g., ‘Oral’ cancer or ‘dental’ trauma-related PSI as well as musculosceletal CBCT-related PSI. Aforementioned PSI reports were excluded. Manual screening of all dentistry-related PSI was performed by an oral and maxillofacial radiologist (MS). Any cases that were questionable regarding their inclusion/exclusion were discussed by all authors. PSI that were generally related to radiology or its ICT environment were included. The data were collected during 10.6.–12.6.2019 (HKI) and 2.7.–4.7.2019 (HUS), and the detailed process is shown in Fig. 2. We decided to include 2012–2014, in addition to 2015–2017, in our evaluated time-period to be able to determine if the reporting activity had grown over the years (Fig. 3). Opting for these years, we were also able to compare the results of an equal time-period before and after oral healthcare was implemented as its own entity in these data systems.

**Table 1:** Keywords and abbreviations used in the search through the HaiPro-data: in addition to searching through the whole HUS- and HKI-data, we also conducted an individual search within the category of medical imaging within the HUS-data using common dental keywords

*All HUS-HaiPro-data	**HUS Kuvantaminen HaiPro-data	***All HKI-HaiPro-data
Hammasröntgen, hammasfilmi, pikkukuva, hf, filmi	Hammas	Hammasröntgen, hammasfilmi, pikkukuv, hf, filmi, röntgenkuv, rtg
OPG, OPTG, PTG	Hampaat	OPG, OPTG, PTG
Leukapanoraamakuva	Hampa	Leukapanoraama
Panoraama, panora	Leuka	Panoraama, panora
BW, bite wing, bitewing, bite, purusiiveke, puru	Leuan	BW, bite wing, bitewing, bite, purusiiveke, puru
Intraor	Leukoj	Intraoraali
LATERAALI, kefalo, kallo, lateraalikallo, kallolateraali		Lateraali, kefalo, kallo, lateraalikallo, kallolateraali, kallokuv
KKTT, kartiokeila		KKTT, kartiokeila
Okklu		Okklu
Suun ja kaulan		Suun ja kaulan
Röntgenputk		Röntgenputk
Sylkikiv, sylkirau		Sylkikiv, sylkirau
		Hammas
		Kuvantami
		Röntgen
		Kaula
		Leuka, leukoj

We compartmentalized all found DMFR-related PSI by their type accordingly to the HaiPro classification system. The event type was categorized to be related to:

1. Laboratory-, medical imaging-, or other patient examination
2. Accident
3. Flow or control of information
4. Medication or fluid therapy (including the use of contrast medium)
5. Medical device or its usage
6. Other treatment or patient monitoring
7. Invasive operation
8. Unknown
9. Undefined.

Furthermore, we assessed the contributing factors of the PSI, which were categorized being related to:

1. Communication and flow of information
2. Patient/customer or relatives
3. Work procedure
4. Work environment (incl. Utensils) and resources
5. Medical devices and equipment
6. Education, instruction about the workplace, and competence
7. No recognizable contributing factors, normal situation
8. Unknown
9. Undefined.

The nature of these main categories was further evaluated and reported in detail, including their risk classification, severity, outcome, and possible corrective actions.

None of the gathered information consists of data that could lead to identifying neither healthcare professionals nor patients.

## Results

**Fig. 4:**
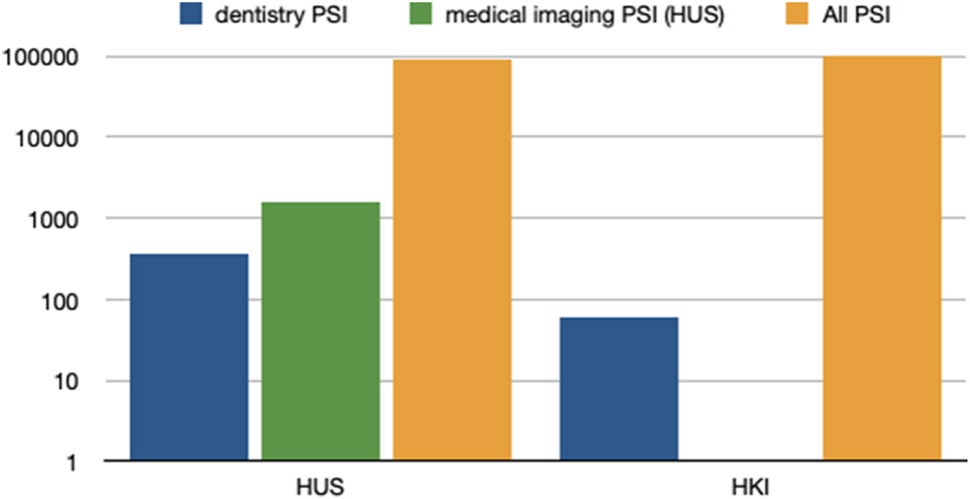
Reported PSI during 2012–2017 (HUS, HKI)

**Fig. 5:**
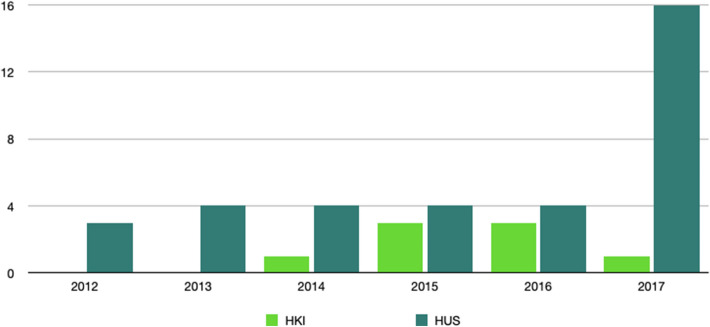
The number of DMFR-related reports in HUS was almost fourfold compared to HKI during the evaluated period

In the HUS and the HKI, a total of 195,593 HaiPro-reports were filed in 2012–2017. In HUS, the total number of these reports was 93,176, of which 1.614 (about 2%) were related to medical imaging and only 359 (about 0.4%) were related to dentistry and in HKI the total amount of the reports was 102,417, of which only 61 (about 0.06%) concerned dentistry (Fig. 4). In the HKI-data, medical imaging-related reports were not available separately categorized comparable to the HUS-data. Altogether, 420 dentistry-related PSI and 2464 PSIs that matched our keyword search, from both HUS and HKI, were evaluated individually not to miss any DMFR-related reports. We found 43 reports of DMFR-related PSIs, of which 35 were from the HUS-data and 8 from the HKI-data (Fig. 5).

In our study, the amount of reported PSIs compared to the number of dental radiographs taken in their corresponding organizations stand for approximately 0.02% in HUS (35/201,113) and 0.001% in HKI (8/729,981).

### Event type and contributing factors

The most prevalent event type of all 43 DMFR-related PSIs was laboratory-, medical imaging-, or other patient examination-related events (14 cases, 33%). The second most common event type of PSI was related to flow or control of information (11 cases, 26%). Other event types included accidents (4 cases), medical devices or their usage (3 cases), medication or fluid therapy (1 case), other treatment or patient monitoring (1 case), and invasive operation (1 case). The event type was defined as unknown on one occasion and left undefined for eight cases. Out of these 14 cases, three reports revealed a mix-up in patients: once a referral to a panoramic image was prescribed to the wrong patient and the error only became clear after the image was obtained. Once a 3D-model for orthognathic surgical planning was delivered under the name of another patient, but this was noticed by the clinician before the event led to any harm to the patient. The third case included a patient receiving another patient’s information regarding the upcoming dates for a scheduled control visit and radiographic examination, but this error was also noticed shortly after it happened, and no harm occurred.

The most common contributing factors linked to the aforementioned event type were perceived as shortcomings related to communication and flow of information (4/14, 29%). Once the contributing factor was reported to be related to medical devices and equipment and once to work procedure, but in more than half of the cases (8/14, 57%), the contributing factors remained unclear as they were either classified as unknown (3 cases) or left completely empty (5 cases).

The second most common event type, total of 11 cases, was defined to be related to flow or control of information and in almost half of these cases, the contributing factor was related to limitations of communication and flow of information (5/11, 45%). Other contributing factors that were identified were related to work procedure (2 cases); education, instruction about the workplace, and competence (2 cases); and to work environment (incl. utensils) and resources (2 cases).

Even though on eight occasions, the event type was not reported, only one of these cases was also left empty regarding information about possible contributing factors. Of the seven remaining cases without a defined event type, the commonest contributing factor was related to medical devices and equipment (3/8, 38%). Other perceived contributing factors were communication and flow of information (2 cases), patient/customer or relatives (1 case), and work environment (incl. utensils) and resources (1 case). Event types revealed contributing factors that were related to communication and flow of information (1 case), patient/customer or relatives (2 cases), work procedure (1 case), medical devices and equipment (1 case), no recognizable contributing factors, normal situation (2 cases), unknown (1 case), or undefined (2 cases).

### Risk classification

Evaluation of the risk classification showed only one report where the risk was assessed to be moderate (risk level III); in all other reported AE, the risk was considered irrelevant (risk level I) or minor (risk level II). On 8/43 occasions, the risk level remained unassessed. This is consistent with the findings stating that mostly no harm or only slight harm had been caused to the patient regarding the outcome of the adverse event. Moderate harm to the patient was reported only once, but the risk level of the event was assessed to be minor (II) even in this case. This report was made by a nurse after a prolonged surgical tooth extraction, pointing out the need for the clinician to better plan the operation, for example using CBCT imaging.

### The outcome for the patient and the organization

When categorized by the contributing factors, the most frequently reported events (12/43) were due to communication problems or varying information gaps that delayed patient care and increased the workload of healthcare professionals. Despite the delay, the AE was evaluated to not having caused any harm to the patient for 75% of the cases (9/12). Out of the remaining 25% occasions, the outcome to the patient was reported as “unknown” once, and on the remaining two occasions, this information was not provided at all. These communication-related AEs were common in the HKI-data as half of all incidents in the HKI-data revealed shortcomings in communication (4/8). Out of all the communication-based problems or information gaps, 25% were related to the failure of identifying the patient correctly and, thus, the radiological examinations were stored under the wrong patient’s data (3/12), but these errors were identified before any harm occurred. While most of the communication problems or information gaps (7/12) were caused by confusion due to insufficient or unclear oral or written communication, almost half of the cases (5/12) revealed that there was a shortcoming in using, finding, or accessing the relevant information by the healthcare workers that would have resulted in avoiding the reported PSI.

The contributing factors for the most reported adverse events included device malfunction (5/43) and organization policies (5/43). Malfunction-related reports were only found in the HUS-data. The malfunction of the device led on all occasions to image loss during radiological examination and thus to the need to renew the examination, which ultimately increased patients’ radiation dose. Most of these device-related reports (80%) were related to a CBCT device. In addition to these five malfunction-related AE, we found two additional CBCT-related PSI in the HUS-data. In one case, the radiographer forgot to change the CBCT imaging protocol settings prior to the examination, leading to the need to renew the radiological examination and the other case was a software-related PSI, which was reported immediately after a scheduled CBCT software update. The update brought about a change in the proportions of the acquired images leading to abnormal measurement results in the acquired data. This problem was quickly noticed on site by the staff of the radiology department and corrected by the device manufacturer.

During ICT-related problems (4/43), radiological images of patients were unobtainable, reportedly delaying patient care. These problems showed no recognizable common variable. Patient-related incidents (4/43) were as common as ICT-related PSI in our data. These were reported due to a highly active child almost running against the panoramic device, while it was being positioned for the examination and due to nausea, dizziness, or fainting of adult patients.

Classified under different event types, we found a total of 8/43 cases where either the radiographs were unattainable by the clinician for various reasons (6/8) or that the report of the patient’s radiograph was unavailable (2/8) at the time of the patient’s appointment with the clinician.

Assessing the outcome for the organization is also part of the HaiPro-report and, in our data, the most common organizational outcome was an increase in the healthcare professionals’ workload (14/43), followed by a decline in the organization’s reputation (9/43). On eight occasions, there was no harm to the organization regarding the AE, and on nine occasions, no assessment of this information was performed. An increase in expenses (2/43) and treatment duration (2/43) was also reported as an adverse outcome for the organization.

## Discussion

Adverse events related to DMFR are rarely reported and the number of reports seems extremely low compared to the number of dental radiographs taken. In 2015, approximately 3.9 million radiological examinations and 1.9 million dental radiographs were obtained in Finland, stating that about 33% of all radiological examinations were related to DMFR [21]. In our data, when we compare the amount of reported PSI related to DMFR (35) and medical imaging (1.614) in secondary care, there is a notable discrepancy between them as DMFR-related PSI represent only about 2% of all medical imaging-related PSI. It is also notable that even though the number of dental radiographs from HUS in comparison to HKI was only about 28% (201,113/729,981), the number of reported PSI from HUS compared to HKI is greater by almost 440% (35/8). These raise the question of whether there are unidentified profession-related (dentistry vs. medicine) or organizational-related factors that explain the aforementioned diverse reporting activity, especially when comparing healthcare professionals working in secondary care to those working in primary care. One possible explanation could be that patient safety culture has a more solid foundation in secondary care. As a secondary care provider, HUS has been actively promoting patient safety over the past decade and it has been a focus of vivid research, for example, the doctoral dissertation of Palojoki S. in 2017 focusing on understanding and thus preventing technology-induced errors in electronic health records, and that of Tolvi M. in late 2020 focusing on the Weekend Effect and Readmissions in HUS [22, 23]. An upsurge in research focusing on patient safety in primary care since 2001 was found by R. Spencer and S. Campbell (2014), although primary care patient safety studies lag behind those for secondary care patients [24, 25]. As HKI followed by HUS, are the two largest employers in Finland and their staff is most likely a quite homogenous group of healthcare professionals regarding their education, working culture, and the use of the same reporting tool for PSI, additional studies are needed to reveal why the reporting activity is considerably lower in primary care. Comparing the qualities of these organizations’ patient safety cultures might also highlight some potentially effective tools for engaging healthcare professionals as active promotors for a better patient safety culture.

This study revealed that patient safety incidents in DMFR are seldom reported and that their outcome to the patient as well as to the organization was mostly considered irrelevant or minor. Our finding of DMFR-related PSI resulting only rarely in severe harm for the patient is consistent with the findings documented by Kasalak Ö. et al. regarding radiology related PSI [26]. Worryingly, even in our small dataset, almost 7% of reported cases revealed a mix-up between patients, which ultimately could lead to severe harm to a patient, especially in secondary care. This finding accentuates the importance of educating oral and other healthcare professionals to use at least two identifiers as stated by WHO when verifying the patient’s identity [27]. This mix-up of patients was also detected by Jämsä J. et al. in their study of differences regarding serious and nonserious PSI reported in the Hospital District of Helsinki and Uusimaa (HUS) [28]. They reported that of the specific types of PSI within serious incidents, the most important was related to laboratory-, medical imaging-, or other patient examination manifesting as samples taken from the wrong patient. In addition, their study revealed that the third most important incident type within the serious incidents was due to equipment malfunction, but their data did not reveal if or how medical imaging devices contributed to this incident group.

In regard to our results, we find that the most important finding of our study is our suspicion of that a considerable number of PSIs regarding DMFR are left unreported by healthcare professionals, although reporting of all noticed PSIs is officially recommended, and even mandatory when related to the use of ionizing radiation in medicine [29]. This also seems to be in accordance with the observation that even after more than a decade since the publication of To Err is Human, a vast majority of all hospital-based errors, accidents, and other adverse events still go unreported by healthcare professionals [30]. In her doctoral thesis in 2016, Hiivala showed that underreporting PSIs and AEs in dentistry has been suspected in numerous preceding publications [31]. Polisena and colleagues (2015) explain that reasons for this vary, as shown by in their systematic review indicating that uncertainty of what should be reported, the fear of punishment, and time constraints are common barriers to the recognition and reporting of adverse events [32]. In Hiivala’s thesis, the reasons for dentists not to report AEs were in accordance with those indicated by Polisena and colleagues [31, 32]. We also question whether the possible AEs are accurately identified and if the reporting process is familiar to the healthcare workers.

In our data, 25% of all reported events were related to CBCT imaging, which could be related to the fact that it is a relatively new imaging modality and a more complex mechanical device in comparison to the more common panoramic and cephalometric imaging devices. This remains unclear, though, and more research is needed to enlighten the patient safety aspects that might be unique to the CBCT imaging modality.

The limitations of this study lie in the relatively short evaluation period, especially as dentistry was implemented as its own entity in the HaiPro databases only recently, in 2015. In addition, the use of a free-text search tool is prone to miss a finding because of typos. To minimize this factor, we executed a systematic and thorough evaluation of all reports that matched our keywords and those related to dentistry in order not to miss any DMFR-related reported patient safety incidents. To the best of our knowledge, patient safety in dentomaxillofacial radiology in combination with differences between primary and secondary care has not yet been studied, and thus, the main strength of this study is its novelty.

Education, as well as clinical audit, emphasizing patient safety related to medical devices in the field of DMFR would probably be useful to inform and to remind healthcare professionals to report patient safety incidents. It might also be beneficial to implement patient safety as its own distinct entity in dental studies, to raise awareness in the field. Although Künzle et al. focused on leadership in critical care teams in their literature review, their conclusion of the pivotal role of effective leaders in promoting team performance and safety raises the question whether this is lacking in dentistry [33]. Finnish dentists work quite independently in primary health care and in the private sector, too, but in secondary care dentists are more commonly part of a team. Perhaps, this and the subsequent lack of leadership explain some of the difference seen in the reporting activity between primary and secondary healthcare, in addition to the aforementioned suspicion of a more solid foundation of the patient safety culture in secondary care. More research is needed to provide information whether DMFR-related AEs are truly left unreported and what are the actual reasons behind the great difference in reporting activity between primary and secondary healthcare workers concerning DMFR.

## Declarations

### Research involves human or animal participant

This article does not contain any studies with human or animal subjects performed by any of the authors.
