# Correction: Patient safety incidents in dentomaxillofacial imaging: reported adverse events from Hospital District Helsinki and Uusimaa and the City of Helsinki during 2012–2017

**DOI:** 10.1007/s11282-023-00709-3

**Published:** 2023-09-02

**Authors:** Marianne Suuronen, Taina Autti, Lasse Lehtonen

**Affiliations:** 1 Department of Dentomaxillofacial Radiology, HUS Medical Imaging Center, Helsinki University Hospital and University of Helsinki, Haartmaninkatu 4, 00290 Helsinki, Finland; 2 Department of Radiology, HUS Medical Imaging Center, Helsinki University Hospital and University of Helsinki, Helsinki, Finland; 3 HUS Diagnostic Services, Helsinki University Hospital and University of Helsinki, Helsinki, Finland

**Correction: Oral Radiology (2022) 39:164–172** 10.1007/s11282-022-00616-z

In this article the wrong figure appeared as Fig. 5; the figure should have appeared as shown below.
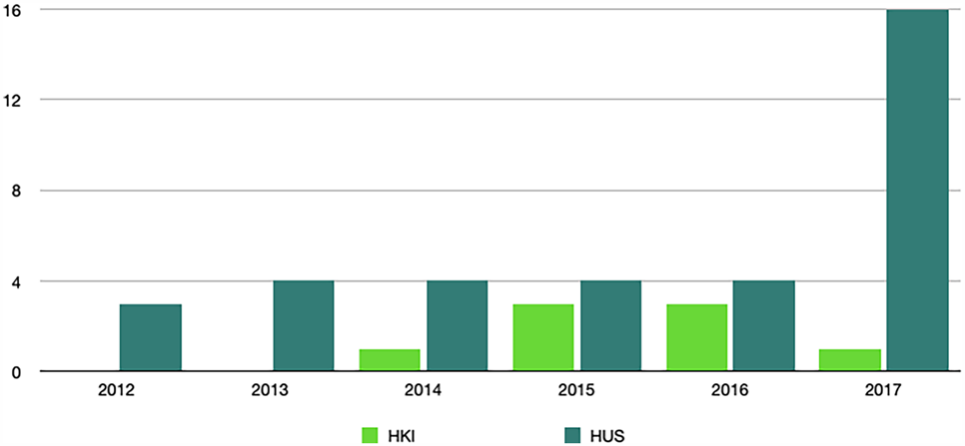


The original article has been corrected.

